# Supplementary material for: The Neural Signatures of Shame, Embarrassment, and Guilt: A Voxel-Based Meta-Analysis on Functional Neuroimaging Studies
Source: Brain Sci. 2023 Mar 26;13(4):559. doi: 10.3390/brainsci13040559 (PMC10136704; doi:10.3390/brainsci13040559)
Supplement: Supplementary file 1 [file brainsci-13-00559-s001.zip › brainsci-2163039-supplementary.pdf]

Table S1. Specific contribution of each study to cluster formation

Analysis	Cluster	Contributors
Shame/embarrassment	1	1 foci from Berthoz et al., 2002<br>1 foci from Finger et al., 2006<br>2 foci from Krach et al., 2011<br>1 foci from Krach et al., 2015<br>1 foci from Laneri et al., 2017<br>1 foci from Mayer et al., 2020<br>1 foci from Michl et al., 2014<br>3 foci from Morita et al., 2012<br>1 foci from Morita et al., 2016<br>1 foci from Morita et al., 2008<br>1 foci from Morita et al., 2014<br>1 foci from Wagner et al., 2011<br>1 foci from Zhu et al., 2018
	2	2 foci from Krach et al., 2011<br>1 foci from Krach et al., 2015<br>1 foci from Laneri et al., 2017<br>2 foci from Mayer et al., 2020<br>1 foci from Morita et al., 2012<br>1 foci from Stroth et al., 2019
	3	1 foci from Melchers et al., 2017<br>1 foci from Morita et al., 2012<br>1 foci from Morita et al., 2016<br>1 foci from Morita et al., 2008<br>1 foci from Morita et al., 2014
	4	1 foci from Morita et al., 2012<br>1 foci from Morita et al., 2016<br>1 foci from Morita et al., 2008<br>1 foci from Morita et al., 2014
	5	1 foci from Krach et al., 2011<br>1 foci from Krach et al., 2015<br>1 foci from Laneri et al., 2017<br>1 foci from Morita et al., 2012
Guilt	1	1 foci from Dominguez et al., 2018<br>1 foci from Fourie et al., 2014<br>1 foci from Gradin et al., 2016<br>1 foci from Molenberghs et al., 2015<br>1 foci from Peth et al., 2015<br>1 foci from Zhu et al., 2018
	2	1 foci from Green et al., 2012<br>1 foci from Kédia et al., 2008<br>1 foci from Takahashi et al., 2004<br>1 foci from Wagner et al., 2011

The table reports the contributors to each cluster.
